# Measuring the impact of the network for improving quality of care for maternal, newborn, and child health: methods, findings, and lessons learned

**DOI:** 10.7189/jogh.15.04201

**Published:** 2025-07-04

**Authors:** Moïse Muzigaba, Theresa Diaz, Sowmya R Rao, Malangizo Mbewe, Martin Dohlsten, Gerard Lopez, Blerta Maliqi

**Affiliations:** 1 Department of Maternal, Newborn, Child, and Adolescent Health and Ageing, World Health Organization, Geneva, Switzerland; 2 Department of Global Health, Boston University School of Public Health, Boston, Massachusetts, USA; 3 Quality Management Directorate, Ministry of Health, Lilongwe, Malawi; 4 United Nations International Children's Emergency Fund, Health & HIV Section, Abuja, Nigeria; 5 Department of Integrated Health Services, World Health Organization, Geneva, Switzerland

## Abstract

**Background:**

The network for improving quality of care for maternal, newborn and child health was established as a partnership of United Nations agencies, implementing partners, and governments in two South East Asian and eight African countries. The methodology that assessed the impact of the network over a five-year period, the results from impact analysis based on selected indicators and the challenges and limitations in collecting and interpreting such data are presented

**Methods:**

The impact analysis was based on three indicators (institutional maternal mortality rate, pre-discharge neonatal mortality, and obstetric case fatality rate) and seven countries. We obtained Relative Risks (RR) and 95% confidence intervals (CI) from Generalized Estimating Equations Negative Binomial models to evaluate the impact of the network initiative on these indicators, unadjusted and adjusted for available facility characteristics.

**Results:**

Most participating facilities were government-managed primary care sites offering antenatal and postnatal care, with prior quality improvement practices. Key factors linked to improved indicators included facility level, geographic location, infrastructure, and partner support. The average rates of institutional stillbirth decreased from the baseline to the intervention period for Ethiopia, Nigeria, and United Republic of Tanzania and increased in Malawi, Sierra Leone and Uganda. Average pre-discharge neonatal mortality rate between the two periods decreased for Ethiopia, Malawi, Sierra Leone, and United Republic of Tanzania but increased for Ghana and Uganda. Average obstetric case fatality rates decreased for Sierra Leone and increased for Ethiopia and Malawi.

**Conclusions:**

Our analysis showed whether the indicators changed over time and to what extent but did not confirm the impact of specific interventions. Quality improvement interventions varied across facilities and between countries.

Globally, progress in reducing maternal mortality has stalled since 2016, with the global maternal mortality ratio (MMR) declining by only 1.5% annually since 2016 [1]. In 2023, an estimated 260 000 women died from pregnancy- or childbirth-related causes, equivalent to one woman every two minutes [1]. There are also large inequalities in maternal mortality by income groups. For example, in 2023, the least developed countries (LDCs) accounted for 43.9% of all maternal deaths, with an estimated MMR of 313 (uncertainty interval (UI) = 277–368), almost 60% higher than the estimated global MMR [1]. Similarly, while some progress has been made in reducing the global stillbirth rate, gains are uneven across settings. Mothers in sub-Saharan Africa and Southern Asia have the highest risk of losing their babies to stillbirths [2]. In 2021, approximately 2.3 million children died during the first month of life, accounting for 47% of the under-five deaths. The highest rates of neonatal mortality are in sub-Saharan Africa, followed by Central and Southern Asia [2].

One main reason for these disparities in maternal and newborn deaths and stillbirths is poor quality-of-care (QoC) [2]. Recognising the need to improve QoC for mothers and newborns and to accelerate the achievement of universal health coverage goals in 2016, the World health Organization (WHO) led a series of meetings and consultations with various stakeholders to discuss what was required to strengthen countries’ health systems to improve QoC [3]. These stakeholders included government representatives, United Nations agencies – including the United Nations International Children's Emergency Fund (UNICEF) and the United Nations Population Fund (UNFPA) – implementing and technical partners, and donors. A facilitated global learning network was conceived as an opportunity to form strategic alliances and generate scalable best practices around QoC implementation, using maternal and newborn health as an entry point. The Network for Improving Quality of Care for Maternal, Newborn, and Child Health (hereinafter referred to as ‘The QoC Network’) was thus formed and launched in 2017 in Lilongwe, Malawi, as a broad-based partnership of 10 committed countries (Bangladesh, Côte d'Ivoire, Ethiopia, Ghana, India, Malawi, Nigeria, Sierra Leone, Uganda, and the United Republic of Tanzania), implementing partners, and funding agencies aiming to implement and sustain QoC at scale based on the principles of equity and dignity [3,4]. The 10 countries committed to reducing maternal and newborn deaths and stillbirths by 50% over five years and to improve user satisfaction with the care received in participating health facilities.

To implement this vision and achieve this ambitious goal, the QoC network articulated four strategic objectives (Leadership, Action, Learning, and Accountability) which describe what must happen at country level to improve quality [4]. These strategic objectives were then translated into a four-component implementation approach (**Figure 1**). The approach articulates key planning and implementation activities recommended at national level, what is needed to support implementation at subnational and health facility levels, various aspects of implementation and impact that should be measured and monitored to assess progress and support accountability and learning, as well as establishing and sustaining a learning system. The third component of this approach, *i.e*. measurement and monitoring implementation and impact, is the focus of this article.

**Figure 1:**
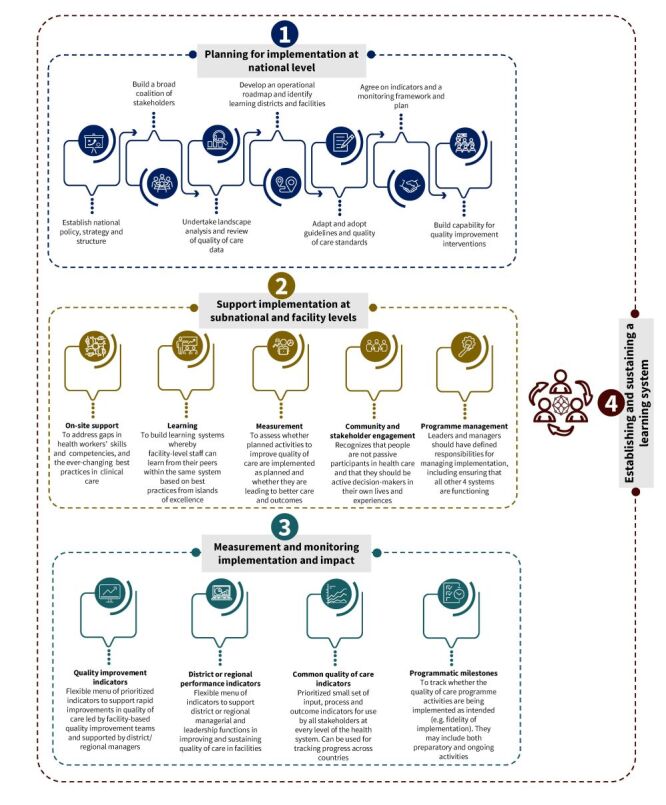
A four-component country implementation approach for the quality-of-care network (adapted from WHO [5] and free to use and modify under CC BY-NC-SA 3.0 IGO).

In this paper, we apply the Standards for Reporting Implementation Studies (StaRI) guidelines [6] to describe how countries in the QoC network were supported by WHO between 2017 and 2023 to operationalise this component. We then provide examples of how specific, time-delimited quality improvement (QI) initiatives were monitored using tailor-made QI indicators and report findings from an outcome and impact analysis of the overall QoC Network initiative, based on a set of three common outcome/impact indicators: institutional maternal mortality rate, pre-discharge neonatal mortality rate, and obstetric case fatality rate. We conclude with a discussion of the implications of the study findings on policy and practice, and offer reflections on key considerations for future impact evaluations of similar multi-level and multi-component QOC initiatives.

## METHODS

### Evaluation design

This was a quantitative impact evaluation of a complex, multi-level, and multi-site QoC initiative using routinely collected data from a set of health facilities purposely selected to participate in the QoC network.

### Interventions to strengthen QoC measurement and monitoring

Prior to the establishment of the QoC network, the practice of measuring QoC for the purpose of improving care was either non-existent, or still in its early stages in many participating countries. At the outset, most countries lacked the foundational technical resources needed for QoC measurement and monitoring, especially resources that were essential for supporting the QI and accountability agenda within the network. To address this gap, the WHO-based QoC Network Secretariat, working through a multi-stakeholder collaboration, was mandated to lead the development and rollout of these resources across network countries. Key resources included tailored QoC measurement guidance and tools for different levels of the health system, as well as a prioritised set of common QoC indicators designed to meet the varied needs of stakeholders.

While some resources could be locally developed to meet country-specific requirements, others were created as global public goods to help countries align with international QoC measurement standards. The Secretariat also established mechanisms for continuous technical support and helped embed common QoC indicators into national health information systems. Implementing and technical partners played a key role in supporting governments to effectively apply both global and locally developed QoC measurement tools in ways that responded to their specific contexts.

#### A common MNH QoC monitoring framework

A QoC monitoring framework for MNH was developed for the network to address QoC measurement and monitoring needs of different stakeholders in the network [7]. This framework includes four ‘buckets’ of QoC indicators and measures which, together, were conceived to provide a holistic view of progress in QoC implementation and the impact of these efforts (**Figure 1**). The monitoring framework also articulates conceptual guidance that can be adapted by country level users rather than prescriptive instructions on what needs to be done to operationalise these indicators and measures. One of the four buckets of indicators is a prioritised small set of common MNH QoC indicators which can be used by all stakeholders to track progress on high level input, processes, outcomes, and impact at different levels of the health system (**Figure 1**). These indicators were measured at the level of the participating health facilities (hereinafter referred to as learning sites) and shared upwards at subnational, national, and global (network secretariat) levels.

#### Interventions to support point of care QI monitoring

The QoC network secretariat also deployed a catalogue of QI indicators at country level designed for use by front line health workers and QI teams to monitor specific QI initiatives [7]. Each learning site could choose which indicator to track from this catalogue given the focus of their QI activity. Furthermore, the QoC Network secretariat developed a Microsoft Excel-based instrument to help learning sites with the systematic documentation of point of care QI efforts for both lateral (between learning sites) and vertical (up the health system hierarchy) sharing of best practices in QI (Template S1 in the **Online Supplementary Document**). The template was modelled on the recommendations from WHO’s programme reporting standards [8] and the structure of WHO’s point of care QI manual [9]. All QI initiatives documented by QoC network countries between 2017 and 2023 were shared with the QoC network secretariat for review, data quality assurance, and global dissemination through a dedicated web-based QI learning hub for the network [10]. They provide a wealth of information on a variety of QI initiatives and evidence of their effectiveness based on monitoring data in the QoC network over a 5-year period.

#### Data collection for common MNH QoC indicators

As explained earlier, the 15 common MNH QoC indicators were proposed to track progress on key processes and outcomes across the QoC Network (Table S1 in the **Online Supplementary Document**). The initial plan was for participating countries to collect these indicators from learning sites and report them through their existing health information systems. However, most of these indicators were new and had not yet been integrated into their national health information systems. In addition, many countries faced significant weaknesses in their data infrastructure which made the measurement and monitoring of these indicators unfeasible at baseline.

To address these challenges, the QoC network secretariat worked with countries to develop an interim QoC data pipeline that provided leeway to countries to start developing or continue strengthening their data systems, while, at the same time, key programmatic activities are being implemented, and data are being used for improvement and decision making. A Microsoft Excel–based tool was developed and distributed to countries to facilitate the collection and reporting of the common MNH QoC indicators from learning sites (Template S2 in the **Online Supplementary Document****)**. The tool was designed for users to enter monthly data elements required to calculate each indicator for both the baseline and intervention periods. Built-in formulas automatically calculated the indicators, serving as a basic quality assurance measure. In countries with less mature data systems, designated QoC focal points at national or subnational levels manually extracted data from health facility records, such as registers. In countries where the relevant data elements were already available in their health information system (*e.g*. DHIS2), data were extracted from those systems and input into the Excel tool. In both cases, the routinely collected data were submitted to the national QoC governance structures at locally determined intervals and shared with the QoC Network Secretariat every six months.

At the Secretariat level, the Excel files were reviewed by a dedicated data manager using Microsoft SQL Server reports to identify data quality issues. Detected errors or inconsistencies were flagged and sent back to countries for correction as much as possible. Once cleaned, the data were uploaded into WHO’s xMart data warehouse through its extract-transform-load process, which conducted further validation checks. The data were then made available for use through JavaScript-based dashboards and reports powered by Microsoft SQL Server views [10]. The cleaner version of this data set was then prepared and used for impact evaluation.

### Impact evaluation

#### Outcomes

To analyse the impact of the QoC network efforts, we used only three common QoC indicators which reflect the goals of the QoC network to ‘reduce maternal and neonatal mortality and stillbirths by 50 percent over a five-year period’ [4]. These indicators are i) institutional maternal mortality rate, ii) pre-discharge neonatal mortality, and iii) obstetric case fatality rate (**Table 1**).

**Table 1:** The metadata of indicators used for impact analysis

Indicator	Definition from the WHO HMIS Guidance	QoC Network Operational Definition	Numerator	Denominator	Data source	Frequency of data collection
Institutional stillbirth rate (disaggregated by fresh and macerated)	Percentage of total institutional stillbirths among all institutional deliveries	Percentage of babies born in a health facility with no signs of life at birth	Number of babies delivered in a facility with no signs of life and born weighing at least 1000 g or after 28 weeks of gestation, per 1000 births (alive or dead at birth)	Number of babies born in the facility (live and stillbirth)	HMIS facility register	Monthly
Pre-discharge neonatal mortality rate	Percentage of total institutional neonatal deaths (28 d or less)	Percentage of babies born live in a facility who die prior to discharge	Number of babies born live in a facility who die prior to discharge from the facility (up to 28 d of completed life), per 1000 live births in a given year or period. This excludes re-admissions for illness.	Number of babies born live in a facility	HMIS facility register	Monthly
Obstetric case fatality rate (disaggregated by direct and indirect when possible)	Percentage of women who delivered at the facility and experienced obstetric complications (regardless of time of onset) and died from these complications before discharge	Percentage of women who delivered at the facility and experienced obstetric complications (regardless of time of onset) and died from these complications before discharge	Number of women who delivered at the facility and experienced obstetric complications (regardless of time of onset) and died from these complications before discharge	Number of women who delivered at the facility	HMIS/facility register	Monthly

HMIS – Health Management Information Systems, QoC – quality of care

#### Targeted sites

We routinely collected data for the three impact indicators along other common MNH QoC indicators from 195 learning sites representing 55 districts and 10 countries in the QoC Network. However, the volume and quality of the data for these indicators varied across learning sites and network countries over the course of implementation. Therefore, the ultimate number of countries and learning sites included in the analysis was much smaller. For example, if a learning site had extreme outliers or significant missing data for a particular indicator during the monitoring period (see impact analysis section), it was excluded from the data set for that indicator. Some countries were excluded from the data set for certain indicators either because the indicator was not yet part of their health information system at baseline, or because it was integrated too late to generate sufficient data for analysis. The five countries that were included in the analysis based on these two criteria included Ethiopia, Ghana, Malawi, Nigeria, and Sierra Leone (Table S2 in the **Online Supplementary Document**).

### Data analysis

#### Analysis of data from point of care QI initiatives

A key component of point of care QI activities at participating learning sites was the selection, monitoring, analysis, and use of databased on one or more QI indicators that were linked closely to a specific improvement aim. Facility-based QI teams selected these indicators either from the QI indicator catalogue developed by the network secretariat, particularly when the QI initiative involved one or more MNH QoC standards [11] or adopted and adapted from other sources such as the existing routine health information system.

The purpose of prioritising these QI indicators was to monitor the effectiveness of the QI activity against the improvement aim, and to make iterative adjustments to the change ideas conceived by the facility-based QI teams to improve care. Data collection occurred at varying intervals (daily, weekly, monthly, and, at most, quarterly) depending on how urgently the data were needed to detect changes in healthcare inputs, processes, and outcomes and to support timely decision-making.

Instead of aggregating data collected over time, QI teams analysed routinely collected QI data using a run chart which is widely used in QI implementation to understand patterns of variation in the data and to determine if the change is an improvement, and whether the QI team is maintaining the gains in health care processes and outcomes. The QI teams used either an automated Microsoft Excel-based run chart template provided by the QoC network secretariat, or other open source or paid automated run chart templates. In learning sites with fewer resources, QI teams plotted routine QI data on a piece of paper which was hung on the wall in the health facility and updated as new data and information were generated from the monitoring process. In most instances, run charts were only built once at least eight data points were available for each indicator, including the baseline monitoring segment. The median values were generated for the baseline and follow-up segments of QI implementation and the run chart rules widely published in the literature were used to analyse and interpret the charts [12].

#### Impact analysis

We performed impact analysis using SAS 9.4 (SAS Institute, Cary, NC, USA) with statistical significance set at a two-sided *P*-value of <0.05. As previously noted, this analysis focused on three indicators: institutional maternal mortality rate, pre-discharge neonatal mortality, and obstetric case fatality rate. The interventions varied by learning site in terms of content, intensity, implementation fidelity, and technical focus. Therefore, the primary aim of this analysis was to assess whether these combined interventions influenced the selected indicators, rather than to determine the effect of any single intervention.

The unit of impact analysis was the country. Each country specified the start date (typically the month) of implementation of QI activities across all learning sites to delineate the baseline and intervention periods. The 12 months preceding the start of QI activities in each learning site were defined as the baseline period, while the period following the 12th month was defined as the intervention period. We created a binary variable to indicate the baseline and the intervention periods. We excluded learning sites with <5 observations at baseline- and/or intervention period for any indicator, learning sites with identical distributions (mean, standard deviation (SD), median, Q1, Q3, minimum and maximum) of baseline- and intervention-period data and learning sites with all summary statistics of 0, 100 (SD = .). The number of learning sites included in the analysis varied by indicator and country, with data aggregated at the country level for each indicator.

We performed regression analyses at the country level to assess the impact of the intervention on each indicator – both unadjusted and adjusted for available facility characteristics. We adjusted all analyses for the clustering of the observations at the facility level. Since the focus was on the average effect of the intervention on the outcomes, we used separate Generalized Estimating Equations (GEE) Negative Binomial models with time (*e.g*. months) (T), indicator for the intervention period (I) and an interaction of time with the indicator (T*I) for each country. We also used the coefficient for the interaction term to determine whether the intervention had a significant impact on the indicator of interest over time. The interaction term was significant only for Uganda and so, for the other six countries we used a model without the interaction term. We then generated least square means with standard errors at both time periods along with the relative risk (RR), 95% confidence intervals (CI), and the *P*-value for the intervention effect. Further, we adjusted all facility characteristics that were available for each country and had sufficient sample size in the regressions. We focused our analyses on complete data and excluded any missing data (Table S2 in the **Online Supplementary Document**).

## RESULTS

### Example of QI monitoring results using run charts

**Figure 2:**
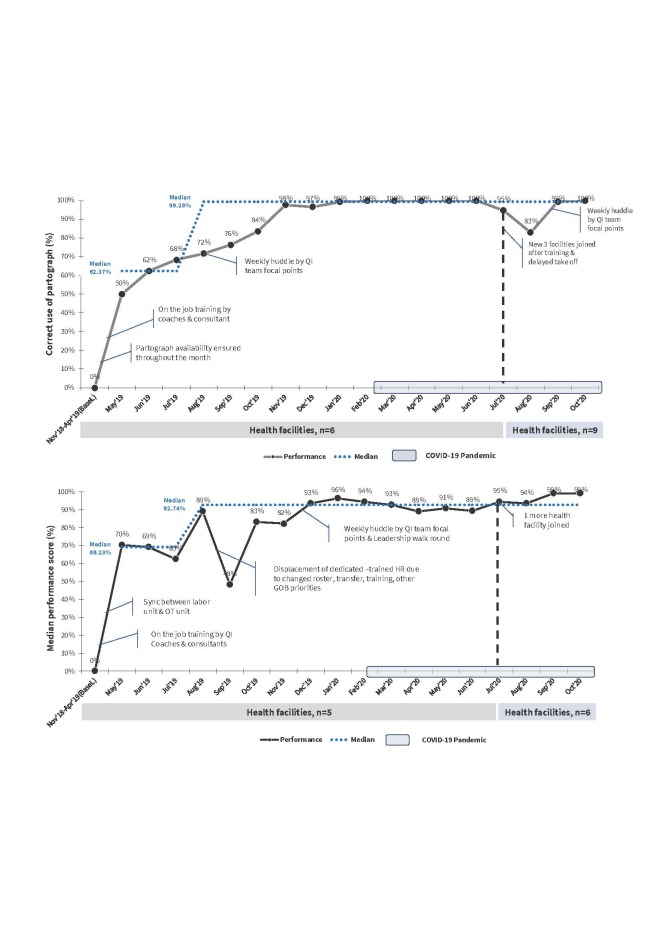
Improvement in the correct use of the partograph and essential newborn care in Manikganj district, Bangladesh.

The two examples of run charts presented in this paper are from one of the countries in the QoC Network [5] (**Figure 2**). They demonstrate how QI teams labelled the charts to demarcate the points at which new change ideas were introduced or old ones modified or removed as part of the QI implementation and performance evaluation process. These examples illustrate how the proportion of partographs used correctly and the median performance score on essential newborn care changed over time across learning sites as various QI activities were implemented. They also highlight the influence of contextual factors, such as COVID-19 pandemic, that may have affected the observed improvements.

#### Descriptive results

The number of observations varied by country, indicator, and intervention period (**Table 2**). On average, almost all countries had a change in the measured indicators. In all countries, most of the learning sites were primary, managed by the government, did not perform c-sections, had been implementing QI work for Maternal, Newborn and Child Health (MNCH) prior to enrolment in QoC network (except Cote d’Ivoire), were supported by other partner organisations, provided postnatal and antenatal care services, teams met regularly to review the data (except Sierra Leone), had water supply in the delivery room, had a functional toilet in the delivery room during and after labour, was part of a referral network and were implementing QI activities in maternal health. While learning sites were mostly rural in Ghana, Malawi, and Sierra Leone, they were mostly urban in Cote d’Ivoire, Ethiopia, and Nigeria (Table S2 in the **Online Supplementary Document**).

**Table 2:** Distribution of observations for mortality indicators by intervention period across learning sites in participating QoC Network countries

Indicator	Period					
**Pre-intervention**			**Intervention**		
**Country Name (No. of learning sites included in the analysis)**	N*	Mean	SD	N*	Mean	SD
Institutional stillbirth rate (total)						
*Ethiopia (32)*	376	30.0	83.8	1030	20.1	39.7
*Ghana (20)*	240	12.4	18.6	950	19.1	86.2
*Malawi (18)*	214	17.3	13.4	233	17.2	15.8
*Nigeria (3)*	23	156.0	334.8	34	42.8	46.7
*Sierra Leone (15)*	180	33.8	47.0	720	28.1	82.2
*Uganda (13)*	148	23.4	32.7	571	70.2	221.5
*United Republic of Tanzania (2)*	24	20.1	7.4	30	23.8	10.6
Pre-discharge neonatal mortality rate						
*Ethiopia (20)*	236	21.2	25.1	659	24.8	79.5
*Ghana (9)*	106	10.8	14.2	429	15.1	18.7
*Malawi (15)*	177	15.8	75.6	197	11.7	15.7
*Sierra Leone (4)*	48	30.6	22.6	192	38.0	73.9
*Uganda (11)*	122	8.0	12.4	393	7.8	12.8
*United Republic of Tanzania (2)*	24	30.5	12.8	30	32.8	19.0
Obstetric case fatality rate (total)						
*Ethiopia (1)*	9	0.5	1.4	16	1.3	2.2
*Malawi (2)*	23	1.6	2.4	18	1.8	3.0
*Sierra Leone (4)*	48	2.6	2.3	190	2.9	3.8

QoC – quality of care, SD – standard deviation

*Number of observations totalled over all the learning sites included in the analysis.

#### Impact-related results

The sub-analyses of the association between facility characteristics and each indicator by country are provided in a supplementary material (Table S3 in the **Online Supplementary Document**). Not all facility characteristics could be included in all the regressions for every country. The key facility characteristics found to be significant included: facility level (primary *vs*. secondary/tertiary), location (urban *vs*. rural), provision of C-sections, prior engagement in MNCH QI work before joining the QoC network, support from other organisations, ongoing implementation of MNH QI activities, regular data review by the facility QI team, availability of at least one handwashing station in the delivery room, access to functional toilets during and after delivery, management type (government *vs*. non-government), provision of neonatal care, and inclusion in a referral network.

**Figure 3:**
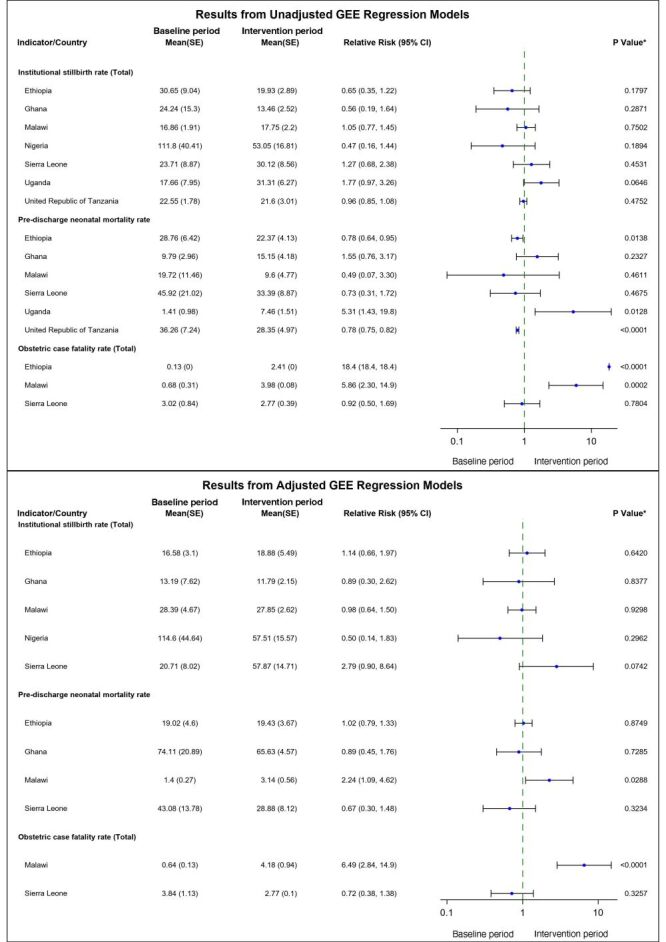
Forest plot showing the impact of multi-faceted, multi-level quality improvement intervention on three impact indicators: Results from adjusted and unadjusted* generalised estimating equation negative binomial regression model. *Depending on the availability of facility characteristics for each country (Table S2 in the **Online Supplementary Document**), models adjusted for level of facility (primary, secondary/tertiary), location (urban/peri-urban, rural), facility perform c-section (no, yes), facility been implementing quality improvement work for maternal, newborn, and child health prior to enrolment in quality-of-care network (no, yes), learning facility supported by other partner organisations (no, yes), team meets regularly to review data (no, yes), facility provide special care for neonates (no, yes), availability of water supply in the delivery room (no, yes), availability of functional toilet to women during labour (no, yes), availability of functional toilet to women after labour (no, yes), availability of at least one functional hand washing facility in the delivery room (no, yes), facility part of a referral network (no, yes), domain of health currently implementing quality improvement activities in (maternal health, newborn health), estimated population size in the catchment area (continuous), annual facility delivery rate (continuous), typical number of deliveries per month (continuous), length (# of months) prior to enrolment in quality-of-care network the facility has been implementing this quality improvement work (continuous), number of times per year facilities receives routine support from the district to build clinical skills (continuous).

Results from the regressions are also displayed as forest plots (**Figure 3**). Results from unadjusted models indicate that average rates of institutional stillbirth decreased between the baseline and intervention periods for Ethiopia (RR = 0.65; 95% CI = 0.35–1.22, *P* = 0.18), Ghana (RR = 0.56; 95% CI = 0.19–1.64, *P* = 0.29), Nigeria (RR = 0.47; 95% CI = 0.16–1.44, *P* = 0.19) and United Republic of Tanzania (RR = 0.96; 95% CI = 0.85–1.08, *P* = 0.47) and increased in Malawi (RR = 1.05; 95% CI = 0.77–1.45, *P* = 0.75), Sierra Leone (RR = 1.27; 95% CI = 0.68–2.38, *P* = 0.45) and Uganda (RR = 1.77; 95% CI = 0.97–3.26, *P* = 0.06), although not statistically significant. As for pre-discharge neonatal mortality rate, the average rates between the baseline and intervention periods decreased for Ethiopia (RR = 0.78; 95% CI = 0.64–0.95, *P* = 0.01), Malawi (RR = 0.49; 95% CI = 0.07–3.30, *P* = 0.46), Sierra Leone (RR = 0.73; 95% CI = 0.31–1.72, *P* = 0.47) and United Republic of Tanzania (RR = 0.78; 95% CI = 0.75–0.82, *P* < 0.0001); the rates increased for Ghana (RR = 1.55; 95% CI = 0.76–3.17, *P* = 0.23) and Uganda (RR = 5.31; 95% CI = 1.43–19.75, *P* = 0.01). Average rates of obstetric case fatality rates decreased for Sierra Leone (RR = 0.92; 95% CI = 0.50–1.69, *P* = 0.78) and increased for Ethiopia (RR = 18.35; 95% CI = 18.35–18.35, *P* < 0.0001) and Malawi (RR = 5.86; 95% CI = 2.30–14.94, *P* = 0.0002).

## DISCUSSION

In this study, we illustrate the use of run charts to analyse trends in QoC measures tailored to specific QI activities. The two examples shown demonstrate how a programme can plot and visualise QI – specific data alongside corresponding QI interventions and help QI teams make the necessary adjustments as needed for the purpose of improving care. Additionally, we assess whether, and to what extent, three common impact indicators which were monitored monthly in the QoC network over a period of five years changed after QI interventions were initiated in a sample of health facilities identified as learning sites. Data from these learning sites were aggregated to constitute a national data set.

Our impact analysis showed that on average, Institutional Stillbirth Rate declined from baseline to intervention periods in Ethiopia Ghana, Nigeria, & United Republic of Tanzania, but increased in Malawi, Sierra Leone, and Uganda, although these changes were not statistically significant. The Pre-discharge Neonatal Mortality Rate decreased for Ethiopia, Malawi, Sierra Leone, and United Republic of Tanzania but the rates increased for Ghana and Uganda. Furthermore, the Obstetric Case Fatality Rate decreased, on average, for Sierra Leone and increased for Ethiopia and Malawi. These results suggest that the impact of the QoC network on the three indicators was not consistent across all countries, with improvements observed in some but not others.

There are several reasons that can explain the divergent findings from this impact analysis. First, there were more than 1000 learning sites that participated in the QoC network over a five-year period. However, findings presented elsewhere [5] show that not all learning sites were able to implement the same package of QI interventions that targeted the three impact indicators of interest, from a theory of change perspective. The timing, duration, content/focus, intensity, dose, and nature of QI interventions varied from one learning site to the other within and between countries. For example, some learning sites spent a good amount of time during the formative stages of implementation working on fixing input- and structure-related QI problems such as renovation of delivery rooms, allocation of additional patient beds, fixing the WASH infrastructure, etc. Other learning sites prioritised process-related QI issues such as ensuring that health care workers can correctly use the partograph or are able to follow WHO clinical guidelines for treatment of specific obstetric complications. The duration of such interventions also varied from 6–24 months. The contribution of QI interventions to programme impact at national level from individual learning sites was not equal. Therefore, it is not surprising that within the context of variable implementation, the impact of the QoC network activities would vary by indicator and across countries, especially considering that our analysis used data aggregated at country level.

It is important to also acknowledge that a range of contextual and external factors beyond the scope of this analysis may have influenced the impact of the QoC network. For example, although not accounted for in the regression models, the COVID-19 pandemic which strained health systems, redirected resources, and negatively impacted service utilisation and quality in many participating countries may have skewed the findings reported here. Other external structural factors not accounted for in our study or elsewhere may include high staff turnover at the level of learning sites, lack of leadership continuity, and chronic health workforce shortages that might have undermined sustained engagement in QI efforts. Although these factors were not captured in the analysis, they represent important limitations and help contextualise the variability in outcomes observed across countries.

Furthermore, this evaluation relied on secondary data collected from learning sites in participating countries, largely through parallel mechanisms that extracted data from countries’ routine health information systems. The maturity and functionality of these systems varied widely across countries and over time, which affected their capacity to consistently generate high-quality data. Some data elements, especially for neonates, were collected using different data sources which complicated data harmonisation from learning sites. Over the five-year period, the data received by the QoC Network Secretariat exhibited variability in completeness and quality across learning sites, time periods, and indicators. Key issues included both random and systematic patterns of missing data, implausible values due to errors in denominators or numerators (*e.g*. obstetric case fatality rate >100%), long sequences of zeros potentially reflecting underreporting rather than true absence of events, and gaps in facility-level covariates due to the use of separate reporting instruments.

These limitations have important implications for interpreting the study findings. First, the exclusion of learning sites with incomplete or poor-quality data may have introduced selection bias, potentially skewing results toward better-performing or better-documented facilities. Second, inconsistencies in data quality across sites and indicators limit the comparability of trends within and between countries. Third, inaccuracies in measurement may have attenuated or exaggerated observed intervention effects, thereby reducing confidence in statistical inferences. Collectively, these issues underscore the importance of strengthening routine health information systems for integrated QoC monitoring and call for cautious interpretation of findings, particularly where data irregularities were most pronounced.

Data quality issues in routine health information systems have been widely documented in similar settings [13–18], including some of the strategies to address them [19]. In the QoC network, both proactive and reactive interventions were implemented by the network secretariat and partners to support countries in collecting and using good quality data locally and sharing the data with the network secretariat as part of the global accountability agenda within the network. Such interventions, though variable in time and intensity across participating countries, included technical support aimed at improving data collection tools and processes, fostering an organisational culture that values data use, and addressing behavioural determinants by motivating health workers and building their capacity to analyse and utilise data effectively. However, the effectiveness of these efforts could not be sustained as they were implemented within a narrow scope focused on QoC rather than being integrated into broader health information system strengthening efforts.

It is also important to note that although, in theory, all learning sites started implementing their QI interventions roughly around the same time (within a theoretical timeframe of three months), which then became the basis for determining the baseline and intervention periods, this may not necessarily have been the case. It is likely that certain learning sites in a specific country had a late start to QI implementation. Variation in impact results is therefore expected if the impact data were averaged across learning sites in a specific country where the baseline and intervention segments overlapped between sites. Unfortunately, for the purpose of this report, sub-analysis at the level of individual learning sites was not feasible to conduct as there were several learning sites, many without enough observations in the two time periods for meaningful site-level analyses.

## CONCLUSIONS

This impact evaluation can be viewed as a natural experiment or a real-world evaluation involving a complex set of QI interventions, implementation approaches, and routinely collected data from multiple sites aggregated at country level. While the QoC network demonstrated the potential to improve MNH outcomes, its overall impact was variable and context-dependent across participating countries included in our analysis. The observed inconsistencies in outcomes are likely attributable to several factors, including significant heterogeneity in the design, implementation, and duration of QI interventions, variation in contextual and health system events such as the COVID-19 pandemic, and limitations in data quality and availability from routine health information systems. Although some countries saw improvements in key indicators, the lack of standardisation in interventions and data limitations undermined the ability to make definitive conclusions about the effectiveness of the QoC network as a whole. Our findings should therefore be interpreted with caution.

Building on these findings, several important implications arise for the future evaluations of similar complex health system initiatives, as well as for policy and practice.

Evaluations of multi-country, multi-site QI initiatives should be designed with sufficient flexibility and granularity to account for the likely variability in implementation fidelity, timing, and contextual factors. These implementation parameters will almost always not be possible to standardise. Therefore, relying solely on aggregated data, particularly when drawn from routine health information systems of varying quality, risks masking meaningful site-level effects or implementation bottlenecks. Future evaluations should therefore prioritise mixed-methods designs, include provisions for site-level or subnational analyses, backed by high-quality, harmonised data systems.

When it is feasible to standardise QI interventions across sites and countries, it is possible to design an impact evaluation that involves routinely collected data sets for the baseline and intervention periods, and for both the implementation sites that receive and those that do not receive QI interventions. Such data sets would make it possible to conduct robust quantitative impact evaluations using difference-in-difference designs or interrupted time series analysis, both of which are known to be strong quasi-experimental evaluation designs for impact analysis [20].

Furthermore, existing data systems would have to be fit-for-purpose to collect high quality data for a set of core indicators (including impact indicators) recognising that not all indicators needed to be tracked and evaluated. The WHO has developed detailed guidance on how countries can achieve this milestone based on their local context [21].

The implementation of QI initiatives at the point of care ought to be more rigorously planned and coordinated. Identifying learning sites with common QoC problems and developing and implementing fit-for-purpose and high impact QI interventions will potentially drive impact better than a set of siloed and heterogenous QI initiatives with variable effects on impact parameters of interest.

In sum, the findings of this evaluation call for a more integrated, adaptive, and context-aware approach to both the implementation and evaluation of similar complex QoC initiatives, especially if they are to be implemented in resource-constrained settings.

## Additional material


Online Supplementary Document
