# Supplementary material for: Measuring the impact of the network for improving quality of care for maternal, newborn, and child health: methods, findings, and lessons learned
Source: J Glob Health. 2025 Jul 4;15:04201. doi: 10.7189/jogh.15.04201 (PMC12580776; doi:10.7189/jogh.15.04201)
Supplement: Online Supplementary Document [file jogh-15-04201-s001.zip › jogh-15-04201-s001.pdf]

Supplement to: Muzigaba M, Diaz T, Rao SR, Mbewe M, Dohlsten M, Lopez G, Maliqi B. Measuring the impact of the network for improving quality of care for maternal, newborn, and child health: methods, findings, and lessons learned. J Glob Health. 2025;15:04201.

Table S2. Maternal and newborn common quality of care indicators for the network

#	Indicator	Definition from the WHO HMIS Guidance	QED Operational Definition	Numerator	Denominator	Data Source	Frequency of Data Collection
**1**	Pre-discharge Maternal deaths	Number of women who delivered in the facility and died prior to discharge	Number of women who delivered in the facility and died prior to discharge	Number of women who delivered in the facility and died prior to discharge	N/A (count indicator)	HMIS/facility register	Monthly
**2**	Pre-discharge Maternal deaths by cause	Number of institutional maternal deaths by cause	Number of institutional pre-discharge maternal deaths by cause (ICD-MM)	Number of maternal deaths by cause (ICD-MM) among women who delivered in the facility and died prior to discharge	N/A	HMIS/facility register	Monthly
**3**	Neonatal deaths in health facilities by cause	Number of institutional neonatal deaths (28 days or less) by cause	Number of institutional pre-discharge neonatal deaths (28 days or less) by cause (ICD-PM)	Number of neonatal deaths by cause (ICD-PM) among babies born live in a facility who die prior to discharge from the facility (up to	N/A	HMIS/facility register	Monthly

				28 days of completed life). This excludes re-admissions for illness.			
**4**	Institutional stillbirth rate (disaggregated by fresh and macerated)	Percentage of total institutional stillbirths among all institutional deliveries	Percentage of babies born in a health facility with no signs of life at birth	Number of babies delivered in a facility with no signs of life and born weighing at least 1,000 grams or after 28 weeks of gestation, per 1000 births (alive or dead at birth)	Number of babies born in the facility (live and stillbirth)	HMIS/facility register	Monthly
**5**	Pre-discharge neonatal mortality rate	Percentage of total institutional neonatal deaths (28 days or less)	Percentage of babies born live in a facility who die prior to discharge	Number of babies born live in a facility who die prior to discharge from the facility (up to 28 days of completed life), per 1000 live births in a given year or period. This excludes re-admissions for illness.	Number of babies born live in a facility	HMIS/facility register	Monthly
**6**	Obstetric case fatality rate (disaggregated by direct and	Percentage of women who delivered at	Percentage of women who delivered at	Number of women who delivered at the facility and	Number of women who delivered at the facility	HMIS/facility register	Monthly

	indirect when possible)	the facility and experienced obstetric complications (regardless of time of onset) and died from these complications before discharge	the facility and experienced obstetric complications (regardless of time of onset) and died from these complications before discharge	experienced obstetric complications (regardless of time of onset) and died from these complications before discharge			
**7**	Pre-discharge counselling for mother and baby	Not applicable	Proportion of women who received pre-discharge counselling for the mother and the baby in a given period	Number of women who received pre-discharge counselling for the mother and the baby in a given period (for minimum elements)	Number of women interviewed who delivered at the facility	Client questionnaire (sample of women)	Quarterly
**8**	Companion of Choice	Not applicable	The proportion of women who wanted and had a companion supporting them during [labor] [childbirth] in	Number of women who wanted and had a companion supporting them during [labor][childbirth] in the health facility	Number of women interviewed who wanted a companion during [labor] [delivery] who delivered at the facility	Client questionnaire (sample of women)	Quarterly

			the health facility				
**9**	Women who experienced physical or verbal abuse anytime during labor, childbirth, or postpartum period	Not applicable	Proportion of women who report physical or verbal abuse anytime during labor, childbirth, or postpartum period. (Physical Abuse: slapped, pinched or punched by a health worker or other facility staff; Verbal Abuse: shouted at, screamed at, insulted, scolded or mocked by a health worker or other staff)	Number of women who report physical or verbal abuse during labor or childbirth	Number of women interviewed who delivered at the facility	Client questionnaire (sample of women) (e.g. exit interview)	Quarterly
**10**	Newborns breastfed within one hour of birth	Percentage of newborns breastfed	Percentage of babies born alive in a facility who	Number of babies born alive in a facility who are breastfed	Number of babies born alive in the facility	HMIS/facility register	Monthly

		within one hour of birth	are breastfed within one hour of birth	within one hour of birth			
**11**	Immediate postpartum Uterotonic for PPH prevention	Percentage of women who gave birth in a health facility who are administered immediate postpartum uterotonic to prevent postpartum hemorrhage	Percentage of women who gave birth in a facility who received a prophylactic uterotonic immediately after birth (ideally within one minute) for prevention of PPH	Number of women who gave birth in a facility who received a prophylactic uterotonic immediately after birth (ideally within one minute) for prevention of PPH	Number of women who gave birth in the facility	HMIS/facility register	Monthly
**12**	Newborns with birthweight documented	Percentage of newborns with documented birthweight in record or register before discharge	Percentage of babies born in a facility in a given period with documented birthweight before discharge	Number of babies born (livebirths and stillbirths) in a facility in a given period with documented birthweight before discharge	Total number of babies born in the facility (livebirths and stillbirths)	HMIS/facility register	Monthly
**13**	Premature babies initiating KMC	Not applicable	Proportion of newborns weighing ≤ 2,000g who are initiated on KMC	Number of newborns weighing ≤ 2,000g who are initiated on KMC (or admitted to	Total number of newborns weighing ≤ 2,000g	HMIS/facility register	Monthly

				KMC unit if separate unit exists)			
**14**	Basic Hygiene Provision	Not applicable	Proportion of QED facilities in which delivery rooms have at least one functional handwashing station with water and soap available	Number of QED facilities in which [all] [at least one] delivery room(s) have at least one functional handwashing station with water and soap available	Number of QED facilities assessed	Facility survey (e.g. district supervision)	Quarterly
**15**	Basic sanitation available to women and families	Not applicable	Proportion of QED facilities with basic sanitation available for women during and after labor and childbirth	Number of QED facilities with basic sanitation available for women during and after labor and childbirth (clean running water, waste disposal facilities, toilets, and sanitation material for women)	Number of QED facilities assessed	Facility survey (e.g. district supervision)	Quarterly

Table S2. Tables of indicators by facility characteristics

	Country Name											
	Ethiopia	Ghana	Malawi	Nigeria	Sierra Leone							
Indicator	Facility Characteristics	Categories	N; Mean(SD)	Median(Q1,Q3)	N; Mean(SD)	Median(Q1,Q3)	N; Mean(SD)	Median(Q1,Q3)	N; Mean(SD)	Median(Q1,Q3)	N; Mean(SD)	Median(Q1,Q3)
CI-4: Institutional stillbirth rate (total)	Level of facility - Combined categories	1. Primary	692;16.92 (62.18)	0.00 (0,22.35)	343;4.58 (12.82)	0.00 (0,0)	298;13.69 (14.17)	10.05 (0,22.06)			180;34.26 (148.29)	0.00 (0,13.71)
		2. Secondary/Tertiary	319;41.5 (28.38)	34.09 (22.85,50.85)	544;31.44 (112.03)	17.47 (0,29.36)	120;24.51 (9.09)	23.97 (17.81,30.48)	57;88.5 (220.12)	34.48 (18.87,47.62)	180;75.87 (47.17)	65.26 (50.09,91.19)
	Geographical location - Combined categories	1. Urban/Peri-urban	637;21.47 (22.05)	18.52 (0,34.09)	430;37.48 (124.94)	20.41 (8.85,32.09)	153;16.52 (11.03)	16.39 (8.2,24.42)	35;58.68 (164.53)	34.09 (20.41,42.02)	180;75.87 (47.17)	65.26 (50.09,91.19)
		2. Rural	418;27.39 (81.57)	0.00 (0,37.04)	457;5.59 (14.88)	0.00 (0,0)	265;16.96 (15.20)	15.27 (0,26.86)	22;135.95 (285.55)	40.31 (14.29,71.43)	180;34.26 (148.29)	0.00 (0,13.71)
	Managing authority - Combined categories	1. Government	1055;23.81 (54.17)	12.82 (0,34.48)	694;9.71 (16.02)	0.00 (0,16.26)	370;16.75 (13.70)	15.85 (4.52,26.04)	57;88.5 (220.12)	34.48 (18.87,47.62)	360;55.06 (111.84)	43.51 (0,69.62)
		2. Private sector/Mission /Faith-based			193;61.82 (183.07)	25.21 (16.13,38.89)	24;26.43 (13.05)	25.81 (18.03,30.93)				
	Does the facility perform C-section procedures?	0. No	487;12.49 (72.63)	0.00 (0,0)	295;3.45 (14.27)	0.00 (0,0)	247;13 (14.70)	9.01 (0,22.06)			180;34.26 (148.29)	0.00 (0,13.71)

		1. Yes	522;34.15 (27.64)	30.38 (14.71,45.45)	652;27.69 (102.77)	15.06 (0,28)	171;22.27 (10.18)	21.49 (15.09,29.41)	57;88.5 (220.12)	34.48 (18.87,47.62)	120;75.35 (54.71)	64.23 (50.19,78.39)
	Has the facility been implementing QI work for MNCH prior to enrollment in QoC network?	0. No	197;13.43 (17.21)	0.00 (0,26.67)	291;10.15 (15.53)	0.00 (0,19.48)	48;17.21 (15.14)	13.32 (0,29.44)	16;92.76 (242.56)	32.53 (20.79,40.81)	240;43.03 (132.50)	0.00 (0,51.41)
		1. Yes	858;26.2 (59.25)	14.49 (0,38.46)	656;24.56 (102.96)	7.77 (0,25.16)	370;16.74 (13.64)	15.96 (4.52,25.64)	41;86.84 (213.92)	36.59 (18.52,49.08)	120;79.13 (39.94)	67.65 (50.32,98.72)
	Is this learning facility supported by other partner organisations (e.g.MCSP, IHI,UNICEF)	0. No	184;11.87 (16.80)	0.00 (0,22.99)	187;9.84 (16.76)	0.00 (0,16.95)	30;10.17 (9.43)	8.32 (3.68,15.46)			180;34.26 (148.29)	0.00 (0,13.71)
		1. Yes	776;27.45 (61.63)	17.10 (0,39.64)	701;21.3 (99.64)	0.00 (0,21.51)	388;17.31 (13.97)	16.92 (4.98,26.38)	57;88.5 (220.12)	34.48 (18.87,47.62)	180;75.87 (47.17)	65.26 (50.09,91.19)
	Does the facility provide special care for neonates?	0. No	209;5.07 (17.86)	0.00 (0,0)	283;2.64 (12.47)	0.00 (0,0)	298;13.69 (14.17)	10.05 (0,22.06)	22;135.95 (285.55)	40.31 (14.29,71.43)	240;46.03 (132.29)	0.00 (0,54.89)
		1. Yes	846;28.45 (58.93)	20.41 (0,39.47)	664;27.59 (101.92)	14.98 (0,28.04)	120;24.51 (9.09)	23.97 (17.81,30.48)	35;58.68 (164.53)	34.09 (20.41,42.02)	120;73.13 (45.69)	66.50 (48.78,90.38)
	Does the facility provide postnatal care services?	1. Yes	1055;23.81 (54.17)	12.82 (0,34.48)	947;20.13 (86.36)	0.00 (0,23.44)	418;16.8 (13.81)	15.85 (4.52,26.04)	57;88.5 (220.12)	34.48 (18.87,47.62)	360;55.06 (111.84)	43.51 (0,69.62)

	Does the facility provide antenatal care services?	1. Yes	1055;23.81 (54.17)	12.82 (0,34.48)	947;20.13 (86.36)	0.00 (0,23.44)	418;16.8 (13.81)	15.85 (4.52,26.04)	57;88.5 (220.12)	34.48 (18.87,47.62)	360;55.06 (111.84)	43.51 (0,69.62)
	Does the team meet regularly to review data?	0. No	160;15.75 (17.85)	11.70 (0,24.17)	108;3.28 (11.07)	0.00 (0,0)	121;18.67 (16.10)	16.95 (0,29.63)			180;58.06 (150.68)	0.00 (0,62.5)
		1. Yes	849;25.19 (59.65)	11.76 (0,35.98)	780;21.05 (94.64)	5.73 (0,21.8)	297;16.03 (12.70)	15.27 (4.93,24.83)	57;88.5 (220.12)	34.48 (18.87,47.62)	120;73.13 (45.69)	66.50 (48.78,90.38)
	How often does the team meet regularly to review data? (Number of times per year)	12	113;7.83 (14.33)	0.00 (0,11.36)	59;38.93 (14.19)	38.92 (29.32,49.79)	120;17.99 (14.35)	19.22 (0,28.41)	57;88.5 (220.12)	34.48 (18.87,47.62)	60;76.91 (26.69)	86.18 (49.22,99.21)
		4			60;6.57 (18.61)	0.00 (0,0)					60;69.35 (58.90)	64.23 (47.22,75.39)
	Is there water supply in the delivery room	0. No	94;25.68 (23.92)	24.70 (0,41.24)			120;14.27 (14.76)	9.90 (0,24.39)				
		1. Yes	915;23.49 (57.54)	10.42 (0,34.09)	888;18.89 (88.97)	0.00 (0,20.53)	298;17.81 (13.29)	17.15 (7.75,26.55)	57;88.5 (220.12)	34.48 (18.87,47.62)		
	What is the main source of water supply?	1. Piped water with tap	638;19.92 (23.27)	14.39 (0,33.33)	779;19.94 (94.73)	0.00 (0,20.88)	298;17.81 (13.29)	17.15 (7.75,26.55)				
		2. Stored water with tap	149;25.97 (126.75)	0.00 (0,10.99)			48;11.89 (14.86)	8.13 (0,21.86)	57;88.5 (220.12)	34.48 (18.87,47.62)		
		3. Stored water without	101;45.66 (48.22)	46.15 (0,81.63)								

		a tap										
		4. No water supply in the delivery room	121;22.46 (23.41)	22.73 (0,34.88)			48;16.92 (15.27)	12.75 (0,28.85)				
	Is there a functional toilet available to women during labour?	0. No	279;16.16 (21.90)	0.00 (0,30.3)	60;13.74 (10.31)	14.60 (4.71,20.45)	169;13.15 (15.40)	9.26 (0,21.74)				
		1. Yes	730;26.57 (63.34)	13.75 (0,37.38)	828;19.26 (92.09)	0.00 (0,20.93)	249;19.27 (12.02)	19.12 (10.37,28.24)	57;88.5 (220.12)	34.48 (18.87,47.62)		
	Is there a functional toilet available to women after labour?	0. No	328;15.04 (22.96)	0.00 (0,27.21)			127;12.91 (14.96)	9.48 (0,21.05)				
		1. Yes	681;27.86 (64.98)	16.67 (0,38.46)	888;18.89 (88.97)	0.00 (0,20.53)	291;18.49 (12.94)	18.69 (8.77,28.17)	57;88.5 (220.12)	34.48 (18.87,47.62)		
	Is there at least one functional hand washing facility in the delivery room?	0. No	308;24.24 (36.01)	0.00 (0,43.48)			48;12.4 (15.65)	8.67 (0,20.73)				
		1. Yes	701;23.45 (61.89)	13.89 (0,33.33)	888;18.89 (88.97)	0.00 (0,20.53)	370;17.37 (13.47)	16.85 (6.99,26.32)	57;88.5 (220.12)	34.48 (18.87,47.62)		
	Is the facility part of a referral network?	0. No			115;1.47 (7.08)	0.00 (0,0)	223;12.99 (14.87)	9.01 (0,22.47)				
		1. Yes	960;24.46 (56.22)	13.16 (0,34.88)	773;21.48 (95.05)	6.33 (0,22.56)	195;21.15 (10.99)	20.47 (13.3,29.21	38;117.77	34.59 (18.87,65.2		

								)	(265.70)	2)		
	In which domain of health are you currently implementing quality improvement activities in your facility?	1. Maternal health	91;37.67 (18.92)	33.71 (23.35,48.03)	549;25.52 (111.96)	8.62 (0,23.08)	394;16.31 (13.88)	15.12 (0,25.5)				
		2. Newborn health			279;8.48 (15.69)	0.00 (0,12.5)	24;24.8 (9.54)	23.41 (16.88,30.82)				
CI-5: Pre-discharge neonatal mortality rate	Level of facility - Combined categories	1. Primary	242;9.8 (19.17)	0.00 (0,13.33)	60;7.02 (7.06)	4.56 (0,11.03)	225;4.4 (7.96)	0.00 (0,7.46)				
		2. Secondary/Tertiary	432;26.94 (52.21)	20.06 (9.2,33.65)	355;17.82 (19.97)	12.08 (0,27.19)	120;25.85 (15.23)	25.09 (16.71,32.33)			180;31.7 (75.42)	25.33 (8.99,39.5)
	Geographical location - Combined categories	1. Urban/Peri-urban	530;20.06 (46.71)	14.22 (0,26.74)	381;16.62 (19.72)	9.90 (0,25.48)	154;13.73 (17.17)	4.74 (0,26.99)			180;31.7 (75.42)	25.33 (8.99,39.5)
		2. Rural	144;23.46 (32.76)	0.00 (0,44)	34;12.23 (7.22)	10.42 (9.09,16.67)	191;10.36 (12.93)	6.76 (0,18.07)				
	Managing authority - Combined categories	1. Government	674;20.79 (44.10)	13.72 (0,27.27)	289;12.71 (19.89)	6.54 (0,16.26)	297;11.35 (14.49)	5.10 (0,20)			180;31.7 (75.42)	25.33 (8.99,39.5)

		2. Private sector/Mission /Faith-based			126;24.41 (13.92)	23.78 (15.5,31.8)	24;27.79 (17.13)	26.79 (15.87,35.41)				
	Does the facility perform C-section procedures?	0. No	48;17.23 (33.71)	0.00 (0,17.24)			173;2.7 (5.71)	0.00 (0,0)				
		1. Yes	580;22.16 (46.34)	16.17 (0,28.71)	415;16.26 (19.05)	10.14 (0,24.92)	172;21.07 (15.88)	20.19 (7.11,30.04)			120;31.41 (91.99)	17.62 (0,38.04)
	Has the facility been implementing QI work for MNCH prior to enrollment in QoC network?	0. No	149;17.27 (10.21)	17.38 (9.3,24.52)	131;19.79 (26.80)	7.04 (0,38.76)	24;2.37 (5.07)	0.00 (0,0)			60;56.19 (125.43)	38.04 (28.22,50.54)
		1. Yes	525;21.78 (49.64)	11.76 (0,30.3)	284;14.63 (13.86)	11.18 (1.89,23.46)	321;12.57 (15.30)	6.76 (0,22.73)			120;19.45 (17.42)	18.62 (0,30.72)
	Is this learning facility supported by other partner organisations (e.g.MCSP, IHI,UNICEF)	0. No	190;20.3 (14.63)	20.04 (11.24,26.92)	60;7.02 (7.06)	4.56 (0,11.03)	30;0.89 (1.82)	0.00 (0,0)				
		1. Yes	438;22.43 (53.62)	11.36 (0,31.06)	296;16.04 (19.99)	9.52 (0,24.69)	315;12.91 (15.33)	7.81 (0,22.95)			180;31.7 (75.42)	25.33 (8.99,39.5)
	Does the facility provide special care for neonates?	0. No					225;4.4 (7.96)	0.00 (0,7.46)			60;6.63 (9.41)	0.00 (0,11.82)

		1. Yes	674;20.79 (44.10)	13.72 (0,27.27)	415;16.26 (19.05)	10.14 (0,24.92)	120;25.85 (15.23)	25.09 (16.71,32.33)			120;44.23 (89.65)	34.24 (22.79,45.45)
	Does the facility provide postnatal care services?	1. Yes	674;20.79 (44.10)	13.72 (0,27.27)	415;16.26 (19.05)	10.14 (0,24.92)	345;11.86 (15.04)	5.35 (0,21.01)			180;31.7 (75.42)	25.33 (8.99,39.5)
	Does the facility provide antenatal care services?	1. Yes	674;20.79 (44.10)	13.72 (0,27.27)	415;16.26 (19.05)	10.14 (0,24.92)	345;11.86 (15.04)	5.35 (0,21.01)			180;31.7 (75.42)	25.33 (8.99,39.5)
	Does the team meet regularly to review data?	0. No	47;25.95 (26.53)	16.06 (4.61,43.29)			96;15.48 (19.69)	6.70 (0,29.38)			60;6.63 (9.41)	0.00 (0,11.82)
		1. Yes	581;21.45 (46.69)	15.15 (0,27.78)	356;14.52 (18.76)	8.75 (0,22.16)	249;10.47 (12.58)	5.21 (0,19.58)			120;44.23 (89.65)	34.24 (22.79,45.45)
	How often does the team meet regularly to review data? (Number of times per year)	12			59;26.78 (17.46)	25.13 (13.75,37.29)	96;15.38 (14.83)	15.17 (0,28.16)			60;32.28 (13.74)	28.94 (21.8,40.25)
		4									60;56.19 (125.43)	38.04 (28.22,50.54)
	Is there water supply in the delivery room	0. No	92;33.05 (23.57)	27.15 (18.55,45.72)			72;3.44 (6.04)	0.00 (0,7.76)				
		1. Yes	536;19.85 (48.01)	13.16 (0,25.98)	356;14.52 (18.76)	8.75 (0,22.16)	273;14.08 (15.90)	8.39 (0,24.31)				
	What is the main source of	1. Piped water with tap	434;17.64 (50.07)	13.24 (0,23.88)	322;14.76 (19.57)	7.81 (0,22.9)	273;14.08 (15.90)	8.39 (0,24.31)				

	water supply?											
		2. Stored water with tap	48;17.23 (33.71)	0.00 (0,17.24)			24;3.18 (4.84)	0.00 (0,7.51)				
		3. Stored water without a tap	54;39.98 (36.32)	37.65 (0,61.22)								
		4. No water supply in the delivery room	92;33.05 (23.57)	27.15 (18.55,45.72)			48;3.57 (6.60)	0.00 (0,7.76)				
	Is there a functional toilet available to women during labour?	0. No	134;32.89 (87.36)	22.47 (7.59,39.51)	60;7.02 (7.06)	4.56 (0,11.03)	95;3.47 (6.71)	0.00 (0,7.3)				
		1. Yes	494;18.77 (23.03)	13.75 (0,26.74)	296;16.04 (19.99)	9.52 (0,24.69)	250;15.05 (16.07)	10.34 (0,25.25)				
	Is there a functional toilet available to women after labour?	0. No	88;33.29 (107.27)	14.76 (2.73,35.96)			77;2.15 (5.65)	0.00 (0,0)				
		1. Yes	540;19.91 (22.91)	15.29 (0,27.84)	356;14.52 (18.76)	8.75 (0,22.16)	268;14.65 (15.73)	9.85 (0,24.67)				
	Is there at least one functional hand washing facility in the delivery room?	0. No	146;35.61 (29.02)	29.63 (15.63,50.25)								
		1. Yes	482;17.6 (48.66)	12.27 (0,23.88)	356;14.52 (18.76)	8.75 (0,22.16)	345;11.86 (15.04)	5.35 (0,21.01)				
	Is the facility part of a referral	0. No					149;2.37 (5.26)	0.00 (0,0)				

	network?											
		1. Yes	628;21.78 (45.49)	15.29 (0,28.71)	356;14.52 (18.76)	8.75 (0,22.16)	196;19.08 (16.03)	18.54 (5.28,29.08)				
	In which domain of health are you currently implementing quality improvement activities in your facility?	1. Maternal health	93;29.93 (27.70)	24.39 (10.93,43.48)	289;10.02 (10.40)	7.81 (0,16.84)	321;11.25 (15.24)	4.05 (0,20.13)				
		2. Newborn health			67;33.93 (30.81)	37.23 (0,55.56)	24;20.03 (8.69)	18.09 (16.99,23.58)				
CI-6: Obstetric case fatality rate (Total)	Level of facility - Combined categories	1. Primary					23;1.2 (2.89)	0.00 (0,0)				
		2. Secondary/Tertiary	25;0.99 (1.95)	0.00 (0,1.43)			18;2.33 (2.10)	1.52 (0.76,3.61)			178;2.8 (3.73)	1.69 (0.89,3.66)
	Geographical location - Combined categories	1. Urban/Peri-urban	25;0.99 (1.95)	0.00 (0,1.43)			18;2.33 (2.10)	1.52 (0.76,3.61)			178;2.8 (3.73)	1.69 (0.89,3.66)
		2. Rural					23;1.2 (2.89)	0.00 (0,0)				
	Managing authority - Combined categories	1. Government	25;0.99 (1.95)	0.00 (0,1.43)			41;1.69 (2.61)	0.00 (0,2.86)			178;2.8 (3.73)	1.69 (0.89,3.66)

	Does the facility perform C-section procedures?	0. No					23;1.2 (2.89)	0.00 (0,0)				
		1. Yes	25;0.99 (1.95)	0.00 (0,1.43)			18;2.33 (2.10)	1.52 (0.76,3.61)			118;3.23 (4.40)	1.71 (0.92,4.44)
	Has the facility been implementing QI work for MNCH prior to enrollment in QoC network?	0. No									59;4.03 (5.64)	2.04 (1.11,5.36)
		1. Yes	25;0.99 (1.95)	0.00 (0,1.43)			41;1.69 (2.61)	0.00 (0,2.86)			119;2.19 (2.01)	1.46 (0.65,3.41)
	Is this learning facility supported by other partner organisations (e.g.MCSP, IHI,UNICEF)	1. Yes	25;0.99 (1.95)	0.00 (0,1.43)			41;1.69 (2.61)	0.00 (0,2.86)			178;2.8 (3.73)	1.69 (0.89,3.66)
	Does the facility provide special care for neonates?	0. No					23;1.2 (2.89)	0.00 (0,0)			59;2.43 (2.42)	1.30 (0.61,4.18)
		1. Yes	25;0.99 (1.95)	0.00 (0,1.43)			18;2.33 (2.10)	1.52 (0.76,3.61)			119;2.99 (4.23)	1.82 (1.03,3.26)
	Does the facility provide postnatal care services?	1. Yes	25;0.99 (1.95)	0.00 (0,1.43)			41;1.69 (2.61)	0.00 (0,2.86)			178;2.8 (3.73)	1.69 (0.89,3.66)

	Does the facility provide antenatal care services?	1. Yes	25;0.99 (1.95)	0.00 (0,1.43)			41;1.69 (2.61)	0.00 (0,2.86)			178;2.8 (3.73)	1.69 (0.89,3.66)
	Does the team meet regularly to review data?	0. No	25;0.99 (1.95)	0.00 (0,1.43)							59;2.43 (2.42)	1.30 (0.61,4.18)
		1. Yes					41;1.69 (2.61)	0.00 (0,2.86)			119;2.99 (4.23)	1.82 (1.03,3.26)
	How often does the team meet regularly to review data? (Number of times per year)	12					18;2.33 (2.10)	1.52 (0.76,3.61)			60;1.96 (1.49)	1.63 (0.75,2.78)
		4									59;4.03 (5.64)	2.04 (1.11,5.36)
	Is there water supply in the delivery room	0. No					23;1.2 (2.89)	0.00 (0,0)				
		1. Yes	25;0.99 (1.95)	0.00 (0,1.43)			18;2.33 (2.10)	1.52 (0.76,3.61)				
	What is the main source of water supply?	1. Piped water with tap	25;0.99 (1.95)	0.00 (0,1.43)			18;2.33 (2.10)	1.52 (0.76,3.61)				
		2. Stored water with tap					23;1.2 (2.89)	0.00 (0,0)				
	Is there a functional toilet available to women during	0. No					23;1.2 (2.89)	0.00 (0,0)				

	labour?										
		1. Yes	25;0.99 (1.95)	0.00 (0,1.43)			18;2.33 (2.10)	1.52 (0.76,3.61)			
	Is there a functional toilet available to women after labour?	1. Yes	25;0.99 (1.95)	0.00 (0,1.43)			41;1.69 (2.61)	0.00 (0,2.86)			
	Is there at least one functional hand washing facility in the delivery room?	1. Yes	25;0.99 (1.95)	0.00 (0,1.43)			41;1.69 (2.61)	0.00 (0,2.86)			
	Is the facility part of a referral network?	0. No					23;1.2 (2.89)	0.00 (0,0)			
		1. Yes	25;0.99 (1.95)	0.00 (0,1.43)			18;2.33 (2.10)	1.52 (0.76,3.61)			
	In which domain of health are you currently implementing quality improvement activities in your facility?	1. Maternal health	25;0.99 (1.95)	0.00 (0,1.43)			41;1.69 (2.61)	0.00 (0,2.86)			

	**Country Name**										
	**Ethiopia**	**Ghana**	**Malawi**	**Nigeria**	**Sierra Leone**						
**Indicator**	**Facility Characteris**	**N**	**Correlation**	**N**	**Correlation**	**N**	**Correlation**	**N**	**Correlation**	**N**	**Correlation**

	tics										
CI-4: Institutional stillbirth rate (total)	Estimated population size in the catchment area (#)	1001	0.1027	888	0.2656	418	0.2259	35	0.1929	360	0.1476
	Annual facility delivery rate (% average) (SKILLED)	566	0.1233	712	-0.0053	244	0.1517	57	0.0298	300	-0.2368
	Typical # of deliveries/month	1011	0.0759	899	0.2910	418	-0.0575	57	0.0122	360	0.1122
	For how long prior to enrollment in QoC network has the facility been implementing this QI work? (months)	867	-0.0005	887	0.0498	370	0.0557	41	-0.2501	60	.
	How often does the facility receive routine support from the district to build clinical skills? (Number of times per year)	874	-0.0471	828	0.1730	322	0.1252	38	0.0813		

CI-5: Pre-discharge neonatal mortality rate	Estimated population size in the catchment area (#)	519	0.1000	351	-0.0459	345	0.4310			180	0.0223
	Annual facility delivery rate (% average) (SKILLED)	178	-0.2164	317	0.1247	195	0.3790			180	0.0668
	Typical # of deliveries/month	628	0.0532	415	0.2552	345	-0.0934			180	0.0658
	For how long prior to enrollment in QoC network has the facility been implementing this QI work? (months)	525	0.0675	351	-0.1977	321	0.1556			60	.
	How often does the facility receive routine support from the district to build clinical skills? (Number of times per year)	384	0.0724	296	0.2909	273	-0.0226				
CI-6: Obstetric	Estimated population	25	.			41	-0.2174			178	-0.1510

case fatality rate (Total)	size in the catchment area (#)										
	Annual facility delivery rate (% average) (SKILLED)	25	.			41	0.2174			178	0.2032
	Typical # of deliveries/month	25	.			41	0.2174			178	-0.1188
	For how long prior to enrollment in QoC network has the facility been implementing this QI work? (months)	25	.			41	-0.2174			59	.
	How often does the facility receive routine support from the district to build clinical skills? (Number of times per year)					41	.				

Table S3. Relative Risks and 95% Confidence Intervals Obtained from Adjusted Models

		Country Name																			
		Ethiopia				Ghana				Malawi				Nigeria				Sierra Leone			
**Indicator**	**Variable**	**RR***	**95% LCL****	**95% UCL****	**P-value**	**RR**	**95% LCL**	**95% UCL**	**P-value**	**RR**	**95% LCL**	**95% UCL**	**P-value**	**RR**	**95% LCL**	**95% UCL**	**P-value**	**RR**	**95% LCL**	**95% UCL**	**P-value**
**Institutional stillbirth rate (total)**	***Indicator for Pre/Post intervention Period***				0.642				0.838				0.930				0.296				0.074
	Post-intervention	1.14	0.66	1.97		0.89	0.30	2.62		0.98	0.64	1.50		0.50	0.14	1.83		2.79	0.90	8.64	
	Pre-intervention	**Ref.**			.	**Ref.**			.	**Ref.**			.	**Ref.**			.	**Ref.**			.
	Level of facility - Combined categories				<0.0001				<0.0001				<0.0001								0.001
	Secondary/Tertiary	2.67	2.41	2.97		2.78	1.68	4.58		2.14	1.92	2.39		.	.	.		2.96	1.52	5.75	
	Primary	**Ref.**			.	**Ref.**			.	**Ref.**			.	.	.	.		**Ref.**			.
	Geographical location - Combined categories				<0.0001				<0.0001				0.435				<0.0001				
	Rural	2.14	2.00	2.28		0.24	0.12	0.47		1.04	0.94	1.14		3.06	2.73	3.42		.	.	.	
	Urban/Peri-urban	**Ref.**			.	**Ref.**			.	**Ref.**			.	**Ref.**			.	.	.	.	
	Does the facility perform C-section procedures?				<0.0001								0.992								
	Yes	7.39	6.32	8.64		.	.	.		1.00	0.60	1.67		.	.	.		.	.	.	
	No	**Ref.**			.	.	.	.		**Ref.**			.	.	.	.		.	.	.	
	Has the facility been implementing QI work for MNCH prior to enrollment in QoC network?				0.518								<0.0001								<0.0001
	Yes	1.66	0.36	7.73		.	.	.		0.70	0.61	0.80		.	.	.		0.96	0.96	0.96	

		Country Name																			
		Ethiopia				Ghana				Malawi				Nigeria				Sierra Leone			
**Indicator**	**Variable**	**RR[*]**	**95% LCL[**]**	**95% UCL[**]**	**P-value**	**RR**	**95% LCL**	**95% UCL**	**P-value**	**RR**	**95% LCL**	**95% UCL**	**P-value**	**RR**	**95% LCL**	**95% UCL**	**P-value**	**RR**	**95% LCL**	**95% UCL**	**P-value**
	No	**Ref.**			.	.	.	.		**Ref.**			.	.	.	.		**Ref.**			.
	Is this learning facility supported by other partner organisations (e.g.MCSP, IHI,UNICEF)?				0.364								0.260								
	Yes	0.78	0.47	1.32		.	.	.		1.17	0.89	1.55		.	.	.		.	.	.	
	No	**Ref.**			.	.	.	.		**Ref.**			.	.	.	.		.	.	.	
	Does the team meet regularly to review data?												0.340								
	Yes	.	.	.		.	.	.		0.87	0.67	1.15		.	.	.		.	.	.	
	No	.	.	.		.	.	.		**Ref.**			.	.	.	.		.	.	.	
	Is there water supply in the delivery room?												0.547								
	Yes	.	.	.		.	.	.		0.92	0.69	1.22		.	.	.		.	.	.	
	No	.	.	.		.	.	.		**Ref.**			.	.	.	.		.	.	.	
	Managing authority - Combined categories								0.464				0.825								
	Private sector/Mission/Faith-based	.	.	.		1.47	0.53	4.09		1.03	0.78	1.37		.	.	.		.	.	.	
	Government	.	.	.		**Ref.**			.	**Ref.**			.	.	.	.		.	.	.	

		Country Name																			
		Ethiopia				Ghana				Malawi				Nigeria				Sierra Leone			
**Indicator**	**Variable**	**RR***	**95% LCL****	**95% UCL****	**P-value**	**RR**	**95% LCL**	**95% UCL**	**P-value**	**RR**	**95% LCL**	**95% UCL**	**P-value**	**RR**	**95% LCL**	**95% UCL**	**P-value**	**RR**	**95% LCL**	**95% UCL**	**P-value**
	In which domain of health are you currently implementing quality improvement activities in your facility?												<0.0001								
	Newborn health	.	.	.		.	.	.		2.37	2.13	2.64		.	.	.		.	.	.	
	Maternal health	.	.	.		.	.	.		**Ref.**			.	.	.	.		.	.	.	
	Is there at least one functional hand washing facility in the delivery room?												<0.0001								
	Yes	.	.	.		.	.	.		1.31	1.15	1.49		.	.	.		.	.	.	
	No	.	.	.		.	.	.		**Ref.**			.	.	.	.		.	.	.	
	Is the facility part of a referral network?												0.2711								
	Yes	.	.	.		.	.	.		0.85	0.63	1.14		.	.	.		.	.	.	
	No	.	.	.		.	.	.		**Ref.**			.	.	.	.		.	.	.	
	Does the facility provide special care for neonates?																				<0.0001
	Yes	.	.	.		.	.	.		.	.	.		.	.	.		0.91	0.91	0.91	
	No	.	.	.		.	.	.		.	.	.		.	.	.		**Ref.**			.

		Country Name																			
		Ethiopia				Ghana				Malawi				Nigeria				Sierra Leone			
**Indicator**	**Variable**	**RR[*]**	**95% LCL[**]**	**95% UCL[**]**	**P-value**	**RR**	**95% LCL**	**95% UCL**	**P-value**	**RR**	**95% LCL**	**95% UCL**	**P-value**	**RR**	**95% LCL**	**95% UCL**	**P-value**	**RR**	**95% LCL**	**95% UCL**	**P-value**
**Pre-discharge neonatal mortality rat**	***Indicator for Pre/Post intervention Period***				0.875				0.729				0.029								0.323
	Post-intervention	1.02	0.79	1.33		0.89	0.45	1.76		2.24	1.09	4.62		.	.	.		0.67	0.30	1.48	
	Pre-intervention	**Ref.**			.	**Ref.**			.	**Ref.**			.	.	.	.		**Ref.**			.
	Level of facility - Combined categories				<0.0001				<0.0001				<0.0001								
	Secondary/Tertiary	2.95	1.85	4.71		0.55	0.55	0.56		11.21	10.31	12.18		.	.	.		.	.	.	
	Primary	**Ref.**			.	**Ref.**			.	**Ref.**			.	.	.	.		.	.	.	
	Geographical location - Combined categories				0.212				<0.0001				<0.0001								
	Rural	1.48	0.80	2.75		3.08	2.87	3.31		0.72	0.71	0.72		.	.	.		.	.	.	
	Urban/Peri-urban	**Ref.**			.	**Ref.**			.	**Ref.**			.	.	.	.		.	.	.	
	Does the facility perform C-section procedures?												<0.0001								
	Yes	.	.	.		.	.	.		0.24	0.23	0.26		.	.	.		.	.	.	
	No	.	.	.		.	.	.		1.00	1.00	1.00	.	.	.	.		.	.	.	
	Has the facility been implementing QI work for MNCH prior to enrollment in QoC network?				<0.001				<0.0001				<0.0001								0.047

		Country Name																			
		Ethiopia				Ghana				Malawi				Nigeria				Sierra Leone			
Indicator	Variable	RR*	95% LCL**	95% UCL**	P-value	RR	95% LCL	95% UCL	P-value	RR	95% LCL	95% UCL	P-value	RR	95% LCL	95% UCL	P-value	RR	95% LCL	95% UCL	P-value
	Yes	0.43	0.26	0.70		0.78	0.78	0.78		1.36	1.36	1.36		.	.	.		0.39	0.15	0.99	
	No	**Ref.**			.	**Ref.**			.	**Ref.**			.	.	.	.		**Ref.**			.
	Is this learning facility supported by other partner organisations (e.g.MCSP, IHI,UNICEF)?				0.005								<0.0001								
	Yes	2.84	1.37	5.88		.	.	.		1285.39	998.98	1653.92		.	.	.		.	.	.	
	No	**Ref.**			.	.	.	.		**Ref.**			.	.	.	.		.	.	.	
	Does the team meet regularly to review data?				0.990								<0.0001								
	Yes	1.00	0.72	1.39		.	.	.		0.86	0.85	0.88		.	.	.		.	.	.	
	No	**Ref.**			.	.	.	.		**Ref.**			.	.	.	.		.	.	.	
	Is there water supply in the delivery room?				0.425								<0.0001								
	Yes	0.99	0.98	1.01		.	.	.		0.95	0.94	0.96		.	.	.		.	.	.	
	No	**Ref.**			.	.	.	.		**Ref.**			.	.	.	.		.	.	.	
	Is there a functional toilet available to women during labour?				<0.0001																
	Yes	0.30	0.19	0.48		.	.	.		.	.	.		.	.	.		.	.	.	

		Country Name																			
		Ethiopia				Ghana				Malawi				Nigeria				Sierra Leone			
**Indicator**	**Variable**	**RR***	**95% LCL****	**95% UCL****	**P-value**	**RR**	**95% LCL**	**95% UCL**	**P-value**	**RR**	**95% LCL**	**95% UCL**	**P-value**	**RR**	**95% LCL**	**95% UCL**	**P-value**	**RR**	**95% LCL**	**95% UCL**	**P-value**
	No	**Ref.**			.	.	.	.		.	.	.		.	.	.		.	.	.	
	Is there a functional toilet available to women after labour?				0.001																
	Yes	2.65	1.27	5.55		.	.	.		.	.	.		.	.	.		.	.	.	
	No	**Ref.**			.	.	.	.		.	.	.		.	.	.		.	.	.	
	Managing authority - Combined categories								<0.0001				<0.0001								
	Private sector/Mission/Faith-based	.	.	.		6.19	6.10	6.29		2.20	2.20	2.20		.	.	.		.	.	.	
	Government	.	.	.		**Ref.**			.	**Ref.**			.	.	.	.		.	.	.	
	In which domain of health are you currently implementing quality improvement activities in your facility?								<0.0001				<0.0001								
	Newborn health	.	.	.		7.31	7.19	7.44		17.37	15.82	19.06		.	.	.		.	.	.	
	Maternal health	.	.	.		**Ref.**			.	**Ref.**			.	.	.	.		.	.	.	
	Is the facility part of a referral network?												<0.0001								
	Yes	.	.	.		.	.	.		1.40	1.38	1.42		.	.	.		.	.	.	
	No	.	.	.		.	.	.		**Ref.**			.	.	.	.		.	.	.	

		Country Name																			
		Ethiopia				Ghana				Malawi				Nigeria				Sierra Leone			
**Indicator**	**Variable**	**RR***	**95% LCL****	**95% UCL****	**P-value**	**RR**	**95% LCL**	**95% UCL**	**P-value**	**RR**	**95% LCL**	**95% UCL**	**P-value**	**RR**	**95% LCL**	**95% UCL**	**P-value**	**RR**	**95% LCL**	**95% UCL**	**P-value**
**Obstetric case fatality rate (Total)**	***Indicator for Pre/Post intervention Period***												<0.0001								0.326
	Post-intervention	.	.	.		.	.	.		6.49	2.84	14.85		.	.	.		0.72	0.38	1.38	
	Pre-intervention	.	.	.		.	.	.		**Ref.**			.	.	.	.		**Ref.**			.
	Does the facility perform C-section procedures?												<0.0001								
	Yes	.	.	.		.	.	.		2.04	1.93	2.16		.	.	.		.	.	.	
	No	.	.	.		.	.	.		**Ref.**			.	.	.	.		.	.	.	
	Has the facility been implementing QI work for MNCH prior to enrollment in QoC network?																				<0.0001
	Yes	.	.	.		.	.	.		.	.	.		.	.	.		0.54	0.47	0.61	
	No	.	.	.		.	.	.		.	.	.		.	.	.		**Ref.**			.

*RR=Risk Ratio; **LCL=Lower Confidence Limit; ***UCL=Upper Confidence Limit
